# Analysis of Natural Clinical Crown Height Changes in Central Incisors and First Molars from Age 8 to 18: A Retrospective Digital Study

**DOI:** 10.3390/jcm14030766

**Published:** 2025-01-24

**Authors:** Luis Huanca Ghislanzoni, Jean Boesiger, Thomas Mourgues, María José González-Olmo, Martin Romero

**Affiliations:** 1 Division of Orthodontics, University Clinics of Dental Medicine, University of Geneva, 1205 Geneva, Switzerland; 2 Division of Fixed Prosthodontics, University Clinics of Dental Medicine, University of Geneva, 1205 Geneva, Switzerland; jean-boesiger@hotmail.com; 3 Department of Orthodontics, Rey Juan Carlos University, 28922 Alcorcón, Spain; pix117@hotmail.fr (T.M.); mariajose.gonzalez@urjc.es (M.J.G.-O.); martin.romero@urjc.es (M.R.)

**Keywords:** dental crown, tooth eruption, maxillofacial development/physiology, models, dental

## Abstract

**Objective:** This thesis aims to digitally study the natural longitudinal evolution of clinical crown height in maxillary and mandibular central incisors and first molars between 8 and 18 years of age. **Methods:** A total of 31 subjects (17 females and 14 males) were selected for the study. Their plaster study models were converted to digital format using a three-dimensional scanner. Digital anatomical landmarks were placed on the central incisors and first molars of all dental arches. The clinical crown height of the central incisors and first molars was then analyzed digitally and longitudinally, based on the position of the marginal gingiva on the vestibular surface of the teeth in question. **Results:** The clinical crown height of maxillary and mandibular central incisors and first molars increased significantly with age (*p*-value < 0.001) from 8 to 18 years. On average, the clinical crown height increased by 1.5 mm for the upper central incisors and 0.8 mm for the mandibular central incisors. The clinical crown height of the upper first molar increased on average by 2.7 mm and 2.1 mm for mandibular first molars. **Conclusions:** The clinical crown height of maxillary and mandibular central incisors and first molars appears to continue increasing beyond 18 years of age. These results suggest that the position of the marginal gingiva on the buccal surface of the teeth is not stable at 18 years of age.

## 1. Introduction

Tooth movements during growth have been studied since the 18th century, beginning with the work of Pierre Fauchard (1728) and John Hunter (1771). Subsequently, the Center for Human Growth and Development at the University of Michigan published “Standards of Human Occlusal Development” (1976), a reference book on the longitudinal study of craniofacial development and tooth movements during growth [1]. Data regarding tooth size, dental arch dimensions, palatal dimensions, and occlusal relationships were measured on cast study models using manual measuring instruments. With the advent of digital technology in dentistry, dentists and orthodontists can now analyze virtual 3D study models more accurately than physical plaster models [2]. Digital measurements have proven to be as reliable and valid as manual measurements [3], and they outperform traditional models by remaining intact over time, offering advantages such as speed, convenience of acquisition, and data preservation over time.

In orthodontics, as in other fields of medicine and dentistry, gingival architecture and disproportionate crown height can negatively affect treatment outcomes, even with perfect alignment of the teeth. The esthetic success of dental or orthodontic treatment is mainly reflected in the golden proportions of the upper anterior teeth, which harmonize the smile with the face [4]. These proportions between the mesiodistal width and the height of the clinical crown largely depend on the position of the gingiva on the vestibular surface of the tooth.

In addition, patients’ demands for aesthetic outcomes have increased considerably, encompassing both dental and gingival criteria. Mucogingival soft tissues play a significant role in smile development [5,6,7,8], with the gingiva representing six of the fourteen fundamental objective criteria of smile aesthetics: gingival health, gingival pocket closure, gingival contour zenith, gingival scallop balance, interdental contact level, and smile symmetry [9]. Beyond these subtle criteria recognized by health professionals, the overall appearance of a smile is largely determined by the amount of exposed soft tissue and the height of the clinical crowns. A gummy smile and/or small clinical crowns are considered to be unaesthetic and affect the attractiveness of a smile [10]. A smile with excessive gum tissue exposure (>4 mm) is generally considered unattractive. Many clinicians perform gingivectomies of the anterior teeth for aesthetic purposes in the final phase of orthodontic treatment. Diode laser gingivectomies and the use of computer programs, such as “digital smile design” (DSD), provide the opportunity to carefully adjust the height/width ratio of the upper teeth, for example, by increasing the clinical crown height in cases with excessive gingival tissue (e.g., gummy smile) [11,12].

The clinical crown height of teeth increases during growth in two phases. First, the active eruption phase extends from the eruption of the tooth into the oral cavity until contact with its antagonist. Secondly, the passive eruption phase extends over several years. This phase is characterized by the apical migration of the gingiva towards the vestibular surface of the anatomical clinical crown until its stabilization approximately 1.2 mm from the enamel–cement junction [13]. More recent studies have demonstrated the influence of the secondary eruption of the maxillary central incisors and first molars on their clinical height increase [14]. It is of essential clinical importance to know at what age the apical migration of the gingiva stabilizes to have a stable clinical crown height [15]. It is not reasonable to perform aesthetic treatments in the anterior areas at an early age until the passive eruption of the teeth is completed. The passive and secondary eruption phases are therefore of great clinical importance in treatment plans for adolescents and young adults who require interventions in anterior areas.

The aim of this research is to measure the longitudinal evolution of the clinical crown height of maxillary and mandibular central incisors and first molars from the age of 8 to 18 years using modern, digital analysis methods.

## 2. Material and Methods

### 2.1. Subjects

This retrospective study was conducted using study models from subjects monitored at the Center for Human Growth and Development (CHGD), University of Michigan, USA. Traditional impressions were taken every 12 to 24 months from ages 3 to 20, followed by casting in plaster. From a database of 967 cases, collected between 1930 and 1967, we selected 31 subjects (17 females and 14 males) based on specific inclusion and exclusion criteria: subjects with complete natural dentition (with the exception of the third molars) without any prosthetic rehabilitation (e.g., implant, removable prosthesis), who have never had orthodontic treatment, with no dental eruption problems (e.g., ankylosis, dental agenesis), with intact plaster casts, and who have been followed up at least four times at the CHGD.

On average, 5.8 maxillary arches and 5.6 mandibular arches per case were analyzed. The teeth examined included the maxillary and mandibular central incisors and first molars, as these are the first to erupt in the oral cavity.

This study is a modern reanalysis of data originally collected in the Michigan Growth Study, a longitudinal research project conducted several decades ago. At the time of the original data collection, there were no established Human Ethics Committees or formalized consent procedures as understood today. Therefore, a Human Ethics and Consent to Participate declaration does not apply to this study.

### 2.2. Digital 3D Analysis of Dental Casts

Beforehand, the physical plaster study models were converted into digital.stl format using a 3Shape R700 scanner (ESM Digital Solution Ltd., Dublin, Ireland).

**Figure 1:**
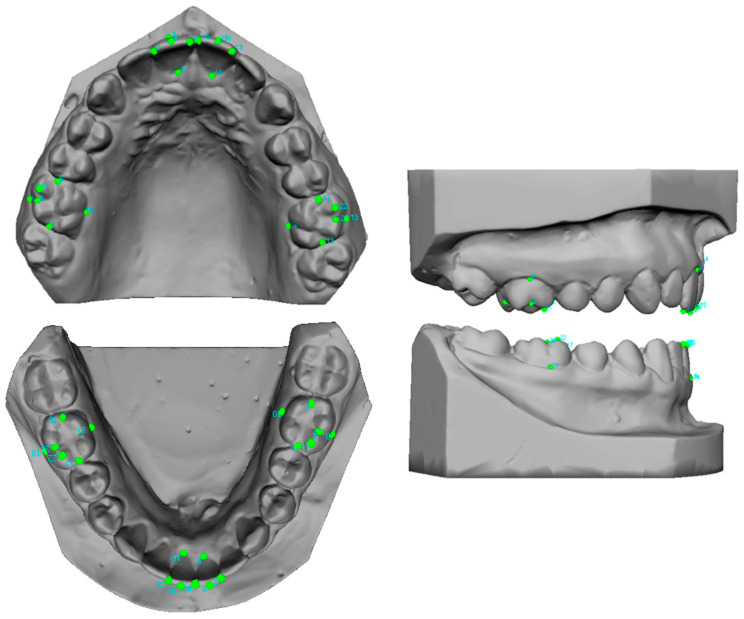
Maxillary and mandibular dental model showing the distribution of 22 points per arch. I green the anatomical landmarks.

In Figure 1, 22 anatomical landmarks (points) were placed on the digital study models of the maxillary and mandibular central incisors (5 landmarks) and first molars (6 landmarks) using VAM software version 2.8.3 (Vectra, Canfield Scientific, Fairfield, NJ, USA). The facial axis of the clinical crown (FACC) is the most prominent portion of the central continuation of the vestibular surface of the incisors and premolars. For molars, the FACC corresponds to the buccal vertical groove that separates the two widest buccal cusps [16]. These markers were placed by the same operator according to the protocol described by Huanca Ghislanzoni et al. [17].

Central incisors:

(1) Five points were placed: the mesial and distal points of the incisal edge, two points corresponding to the gingival and incisal limits of the vestibular axis of the clinical crown (vestibular FACC), and one point corresponding to the gingival limit of the palato–lingual axis of the clinical crown (palato-lingual FACC) corresponding to the continuation of the vestibular FACC on the palatal/lingual surface [17].

First molars:

(2) Six points were placed: the mesial and distal points of the occlusal surface, and two points corresponding to the gingival and occlusal limits of the vestibular axis of the clinical crown (vestibular FACC). The occlusal point is positioned between the two widest cusps, and the gingival point is positioned at the gingival limit of the vestibular groove. One point corresponds to the gingival limit of the palatolingual axis of the clinical crown (vestibular FACC), the lingual axis of the clinical crown (palato-lingual FACC), and a sixth point is added at the apex of the maximum convexity of the occlusal surface of the mesio-vestibular cusp.

**Figure 2:**
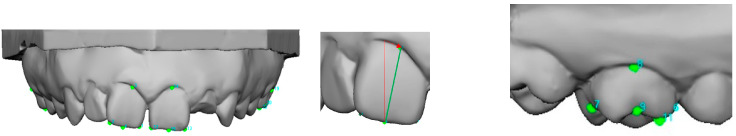
Clinical crown height (in green) measured according to the FACC of the central incisor and of the first molars. In red: clinical crown height measured from the most apical point of the gingival concavity and the incisal edge.

In this study, clinical crown height is assessed using the distance in millimeters from the FACC. For central incisors, clinical crown height is measured digitally in millimeters between the gingival point of the FACC and the incisal point of the FACC. For first molars, the clinical crown height is measured digitally in millimeters between the gingival point of the FACC and the occlusal point of the FACC. In doing so, the clinical crown height is measured along the mesio–distal axis of the tooth (Figure 2). As a reference, previous studies assessed the clinical crown height of central incisors based on the most apical point of the gingival concavity to the incisal edge.

**Figure 3:**
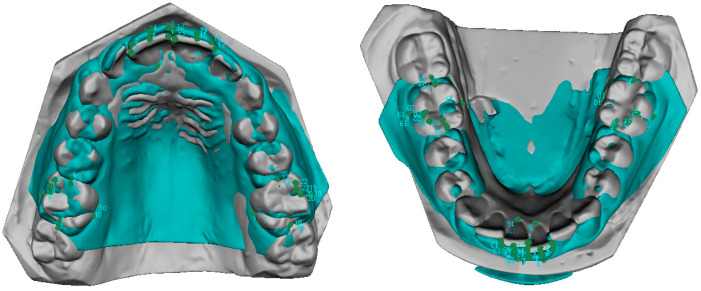
Superimposition of a maxillary model with its associated reference model. Superimposition of a mandibular model with its associated reference model.

To verify the reproducibility and consistency of the points [18], the operator determines a maxillary model and a mandibular reference model for each case, using the oldest models available. The operator then superimposes each maxillary model with the reference maxillary model using the 22 common markers (5 markers per central incisor and 6 markers per first molar) to create a similar reference plane and reduce error (Figure 3). The same procedure is used for the mandibular models.

To evaluate measurement errors, the selection of anatomical landmarks (points) was repeated after 4 weeks by the same operator on 5 randomly selected digital models.

### 2.3. Statistical Analysis

Statistical analysis was performed using SPSS version 24 (SPSS Inc., Chicago, IL, USA). Measurement errors were calculated using Dahlberg’s D formula. Student’s t-test for paired data were used to identify any systematic method error. Means and standard deviations were calculated for all measurements at T1, T2, and T3. For tooth measurements, the mean of both sides was used. Kolmogorov–Smirnov tests showed a normal distribution for all variables, and changes between phases were evaluated with repeated measures analysis of variance. A linear mixed-effects regression model was performed to determine the effects of jaw, tooth, and side nested within the subject. The significance level was set at 0.05.

## 3. Results

To exclude possible systematic measurement errors, the Luis Huanca protocol was used [17]. The was calculated according to Dahlberg’s formula and it corresponded to 0.13 mm.

The results show that the clinical crown height of all teeth increases significantly with age as shown by the linear regression model.

### 3.1. Central Incisors

**Figure 4:**
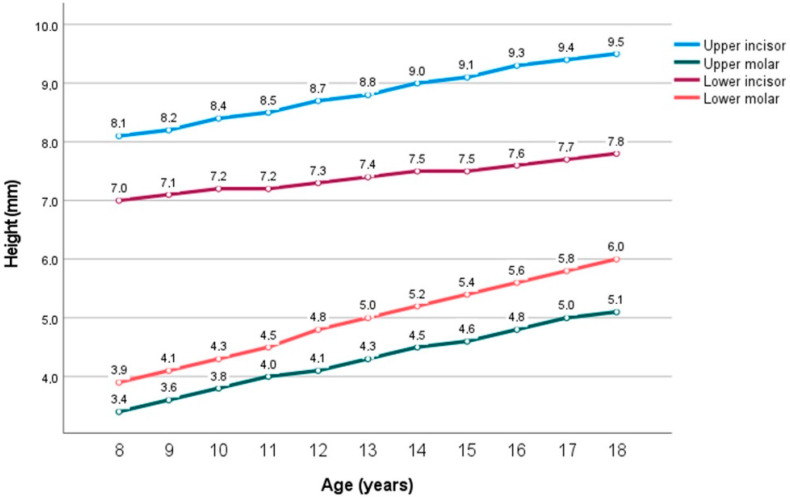
Average clinical crown heights of upper and lower central incisor crowns and upper and lower first molar crowns.

As shown in Figure 4, the height of the incisors increases with age, although at different rates. For example, the mandibular incisors increase less rapidly, and their height difference with the maxillary incisors increases with age.

At age 8, the mean heights are 8.1 mm, 7 mm, 3.9 mm, and 3.4 mm for the maxillary incisor, mandibular incisor, mandibular molar, and maxillary molar, respectively.

Mandibular incisors at age 8 have a clinical crown height of 7 mm, increasing by 0.08 mm on average until age 18. Maxillary incisors at age 8 have a clinical crown height of 8.1 mm, increasing by 0.15 mm on average until age 18.

### 3.2. First Molars

The upper and lower molars grow in height faster than the incisors. However, maxillary molars have the lowest clinical crown height during growth.

In general, maxillary molars are similar in height to mandibular molars 4 years earlier. At age 12, the height of the maxillary molars (4.1 mm) is similar to that of the mandibular molar at age 9. At age 13, the height of the maxillary molars (4.3 mm) is similar in height to that of the mandibular molar at age 10. At age 14, the height of the maxillary molars (4.5 mm) is similar to that of the mandibular molar at age 11. At 17 years, the height of the maxillary molars (5 mm) is similar to that of the mandibular molar at age 13 (5 mm).

Mandibular molars at age 8 have a height at the clinical crown height of 3.9 mm, increasing by 0.21 mm on average until age 18. Maxillary molars at age 8 have a clinical crown height of 3.4 mm, increasing by 0.17 mm on average until age 18.

A linear mixed-effects regression model was calculated (Table 1). The average coefficient of the lines represents the average annual increment of each type of tooth, while the first value of the equation represents the y-intercept of the regression line, reflecting the baseline value of crown height.

Mandibular incisor: Height = 7.0 + 0.08 × Age
Mandibular molar: Height = 3.9 + 0.21 × Age
Maxillary incisor: Height = 8.1 + 0.15 × Age
Maxillary molar: Height = 3.4 + 0.17 × Age

## 4. Discussion

The results show that the clinical crown height of the central incisors and first molars increases significantly with growth. This increase is partly due to the secondary eruption of the teeth. During adolescence, a decrease in the probing depth of the teeth is observed, resulting in an apical movement of the vestibular gingiva over the tooth surface and an increase in the height of the clinical crown. Furthermore, an association between increased lower facial height during growth, gingival displacement of the gingiva, and secondary eruption of the teeth has been demonstrated [14,15]. These same results have been found in other research carried out by Natsmeda et al., which showed that the clinical height of the upper incisor crown increases while the width of the mesiodistal crown decreases from 13 to 17 years of age [19].

L.A. Morrow et al. studied the longitudinal evolution of clinical length from the age of 12 to 19 years. Their results showed that the process of passive eruption continues during the adolescent years, resulting in an increase in the height of the clinical crown of teeth, with a significant difference in clinical crown height between boys and girls [20]. Their results are similar to ours in terms of the speed of passive eruption of teeth: the height of mandibular central incisors increases more slowly compared to the height of maxillary lateral incisors, maxillary central incisors, and maxillary canines. Furthermore, our results confirm the observations of L.A. Morrow et al. on the clinical crown height of the lower central incisors, which continue to erupt passively until at least 18 years of age. Previously, Volchansky and Cleaton-Jones stated that the crown height of the lower central incisors stabilized by the age of 10 [21]. Similarly, in the study by Massaro et al., the clinical crown of the upper molar was observed to increase at a slower rate compared to other teeth, which is consistent with our findings. However, while the author reports an increase of 0.01 mm between the ages of 13 and 17, this research records an increase of 0.7 mm. This discrepancy could be attributed to the measurement method used [22].

Regarding the mean heights of the upper and lower central incisors at 18 years, our results show mean height values 0.5 mm lower, an estimate similar to the results of Volchansky et al. (1981) [21]. This may be explained by the fact that previous studies measured this distance based on the most apical point of the gingival concavity and the apex of the incisal edge. Our measurements, based on the FACC (Figure 2), may be less than or equal to the distance between the most apical point of the gingival concavity and the apex of the incisal edge [19,23]. This results in mean clinical crown heights that may be significantly lower for maxillary and mandibular central incisors. However, our measurements reflect the clinical crown height along the true mesiodistal axis of the tooth. Basing our measurements on the FACC allows us to maintain a fixed anatomical reference point for digital measurements and avoids the need to reposition anatomical reference points randomly, increasing the accuracy of longitudinal measurements. On the other hand, it is possible that natural wear of the incisal edges altered the clinical results of the crown height of the central incisors. Models with extreme wear were excluded from the study. In addition, the models were previously studied manually by researchers at the University of Michigan using calipers, potentially causing iatrogenic wear on the study models and thus altering the measurements.

The measurement of molar height is not affected by natural wear because it is taken according to the FACC of the molar, which corresponds to the vestibular vertical sulcus separating the two widest cusps. Since natural wear of molars occurs mainly on the medial side of the palatal cusps of maxillary teeth and on the medial side of the buccal cusps of mandibular teeth [24], clinical measurements of molar crown height are not influenced by natural wear.

Dental models with indirect restorations, such as crowns or veneers, were excluded from the study. However, models with direct or indirect restorations like amalgams, composite resins, and inlays restoring Class I and II black cavities in molars were included, provided the buccal surface remained intact. Additionally, direct restorations of Class III black cavities in incisors were allowed. Models were excluded if they showed gingival swelling due to poor oral hygiene and inflammation. However, the effect of mild to moderate gingivitis on gingival position cannot be examined in plaster models.

Evaluating the mean values of clinical crown height as a function of age (Figure 1), we hypothesize that the increase in clinical crown height of maxillary central incisors slows down with age. Between the ages of 8 and 15 years, the height increases by approximately 0.2 mm per year; from 15 to 18 years, the increase slows to about 0.1 mm per year. Future research should continue beyond the age of 18 to assess whether this increase stabilizes, which would be clinically important for aesthetic pre-treatments in young adults. For mandibular central incisors, the increase in clinical crown height, although small, appears constant from ages 8 to 18, similar to mandibular first molars.

From this research, we can state that tooth height does not stabilize the vestibular marginal gingival position between 8 and 18 years in the teeth studied. Consistent with other studies [20,22,23,25], we cannot confirm that these teeth have completed their passive eruption by age 18, as this process continues beyond that age. The position of the gums, the height/width ratio of the teeth, the zenith of the gingival contour, and the symmetry of the gingival contour are essential elements in the aesthetics of a smile and the success of treatment. Diode laser gingival manipulation is often performed during or after orthodontic treatment to meet these aesthetic expectations [26,27]. In adolescents, it is important to remember that passive tooth eruption is not complete. It is difficult to determine an ideal age for gingival recontouring.

## 5. Conclusions

The apical movement of the gingiva over the teeth during growth is a known phenomenon. This study confirms the significant increase in clinical crown height with growth in maxillary and mandibular central incisors and first molars at a pace ranging from 0.1 to 0.2 mm per year between the ages of 8 and 18 years.

## Figures and Tables

**Table 1:** Mixed effects linear regression model. Crossed effects jaw, tooth, and side are nested within the subject. Subject has a random effect on slope.

	Estimate	Std. Error	t. Value	p.z
Intercept *	7.01	0.14	51.94	<0.001
Age	0.08	0.01	7.41	<0.001
Maxillaire = 1	1.06	0.11	9.90	<0.001
Tooth = 6	−3.12	0.15	−20.60	<0.001
Age:Maxillaire = 1	0.07	0.01	6.54	<0.001
Age:Tooth = 6	0.14	0.01	12.55	<0.001
Maxillaire = 1:Tooth = 6	−1.51	0.09	−16.96	<0.001
Age:Maxillaire = 1:Tooth = 6	−0.11	0.01	−7.67	<0.001

* = Height mean value for a mandibular-tooth 1 at 8 years of age.

## Data Availability

The data that support the findings of this study are available on request from the corresponding author. The data are not publicly available due to privacy and ethical restrictions.
